# Bis{2-[imino­(phen­yl)meth­yl]-5-meth­oxy­phenolato-κ^2^ *N*,*O* ^1^}nickel(II)

**DOI:** 10.1107/S1600536812043061

**Published:** 2012-10-20

**Authors:** Yu Xiao, Zhong-Qiu Li, Xue Yan Peng

**Affiliations:** a College of Environmental Science and Engineering, Guilin University of Technology, Guangxi Key Laboratory of Environmental Engineering, Protection and Assessment, Guilin 541004, People’s Republic of China

## Abstract

The title complex, [Ni(C_14_H_12_NO_2_)_2_], lies about an inversion center. The Ni^II^ atom is coordinated in a slightly distorted square-planar geometry by two O atoms and two N atoms from two 2-[imino­(phen­yl)meth­yl]-5-meth­oxy­phenolate ligands. The dihedral angle between the symmetry-unique phenyl and benzene rings is 73.2 (1)°.

## Related literature

For background to 2-imino­(meth­yl)phenol compounds, see: Zhang *et al.* (2008 ▶, 2009 ▶); Jiang *et al.* (2003 ▶); Liu *et al.* (2009 ▶). For a related structure, see: Bernès (2010) ▶.
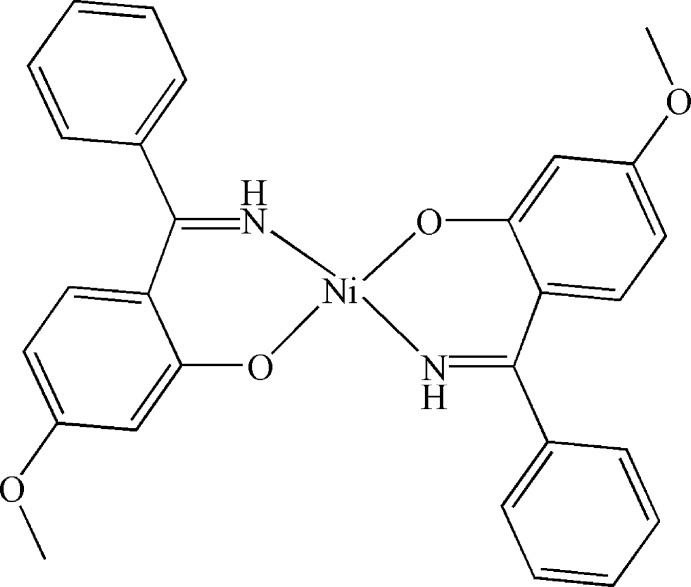


## Experimental

### 

#### Crystal data

[Ni(C_14_H_12_NO_2_)_2_]
*M* *_r_* = 511.20
Monoclinic, 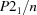

*a* = 11.882 (2) Å
*b* = 5.4983 (10) Å
*c* = 17.494 (3) Å
β = 91.913 (2)°
*V* = 1142.3 (4) Å^3^
*Z* = 2
Mo *K*α radiation
μ = 0.89 mm^−1^
*T* = 296 K
0.24 × 0.15 × 0.10 mm

#### Data collection

Bruker SMART CCD diffractometer
Absorption correction: multi-scan (*SADABS*; Bruker, 2001 ▶) *T*_min_ = 0.244, *T*_max_ = 0.453
5526 measured reflections
2010 independent reflections
1680 reflections with *I* > 2σ(*I*)
*R*_int_ = 0.021

#### Refinement

*R*[*F* ^2^ > 2σ(*F* ^2^)] = 0.025
*wR*(*F* ^2^) = 0.064
*S* = 1.03
2010 reflections
161 parameters
2 restraints
H-atom parameters constrained
Δρ_max_ = 0.19 e Å^−3^
Δρ_min_ = −0.15 e Å^−3^

### 

Data collection: *SMART* (Bruker, 2001 ▶); cell refinement: *SAINT* (Bruker, 2001 ▶); data reduction: *SAINT*; program(s) used to solve structure: *SHELXS97* (Sheldrick, 2008 ▶); program(s) used to refine structure: *SHELXL97* (Sheldrick, 2008 ▶); molecular graphics: *SHELXTL* (Sheldrick, 2008 ▶); software used to prepare material for publication: *SHELXTL*.

## Supplementary Material

Crystal structure: contains datablock(s) I, global. DOI: 10.1107/S1600536812043061/lh5543sup1.cif

Structure factors: contains datablock(s) I. DOI: 10.1107/S1600536812043061/lh5543Isup2.hkl

Supplementary material file. DOI: 10.1107/S1600536812043061/lh5543Isup3.cdx

Additional supplementary materials:  crystallographic information; 3D view; checkCIF report

## Figures and Tables

**Fig. 1.**
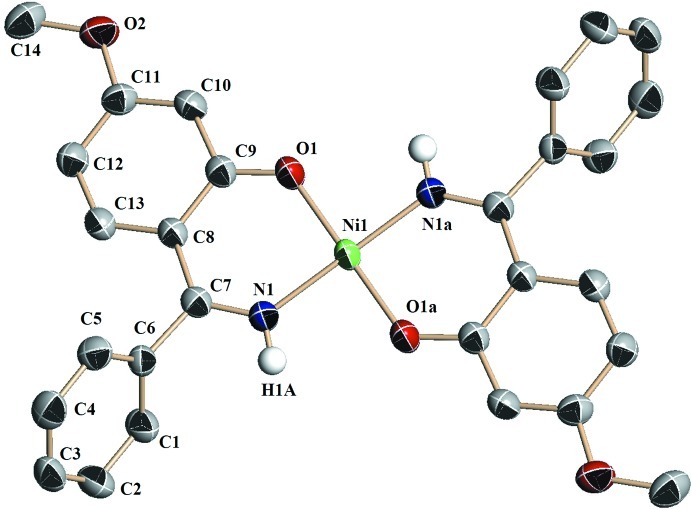
The molecular structure of (I), showing 30 % probability displacement ellipsoids. H atoms bonded to atoms are not shown. Symmetry code (a); 1-x, -y, 2-z.

## supplementary crystallographic information

### Comment

2-Imino(methyl)phenol compounds have been studied for many years (Jiang *et al.*, 2003; Zhang *et al.*, 2008, 2009; Bernés 2010; Liu *et al.*, 2009) and have attracted interest because of their magnetic properties. The crystal structure of the title compound (I) is presented herein.

The molecular structure of (I) is shown in Fig .1. The Ni^II^ ion lies on a centre of inversion and is coordinated by two O atoms and two N atoms from two bidentate ligands, forming a slightly distorted square-planar geometry. The dihedral angle between the symmetry unique phenyl and benzene rings is 73.2 (1) °.

### Experimental

Complex (I) was prepared from a mixture of 2-hydroxy-4-methoxy benzophenone (1 mmol, 0.228 g), ammonia (25%, 0.5 ml), triethylamine (0.5 ml), nickel(II) acetate tetrahydrate (0.5 mmol, 0.127 g) and methanol(8 mL) sealed in a 15 mL teflon-lined stainless steel bomb, and kept at 393 K for 120 h under autogenous pressure. After the reaction was slowly cooled to room temperature, green rectangular plates were produced (yield: 63%, based on Nickel). Anal. Calcd for C_28_H_24_N_2_NiO_4_(%): C, 65.78; H, 4.73; N, 5.48. Found(%): C, 65.72; H, 4.76; N, 5.53.

### Refinement

H atoms were positioned geometrically and refined with a riding model, with distances 0.86(N—H), 0.96(CH_3_) or 0.93 Å (aromatic ring), and with *U*_iso_(H) = 1.2 *U*_eq_(aromatic ring, N—H) or *U*_iso_(H) = 1.5 *U*_eq_(CH_3_).

### Crystal data

**Table:**

[Ni(C_14_H_12_NO_2_)_2_]	*F*(000) = 532
*M**_r_* = 511.20	*D*_x_ = 1.486 Mg m^−^^3^
Monoclinic, *P*2_1_/*n*	Mo *K*α radiation, λ = 0.71073 Å
Hall symbol: -P 2yn	Cell parameters from 2010 reflections
*a* = 11.882 (2) Å	θ = 2.0–25.0°
*b* = 5.4983 (10) Å	µ = 0.89 mm^−^^1^
*c* = 17.494 (3) Å	*T* = 296 K
β = 91.913 (2)°	Plate, green
*V* = 1142.3 (4) Å^3^	0.24 × 0.15 × 0.10 mm
*Z* = 2	

### Data collection

**Table:**

Bruker SMART CCD diffractometer	2010 independent reflections
Radiation source: fine-focus sealed tube	1680 reflections with *I* > 2σ(*I*)
Graphite monochromator	*R*_int_ = 0.021
φ and ω scans	θ_max_ = 25.0°, θ_min_ = 2.0°
Absorption correction: multi-scan (*SADABS*; Bruker, 2001)	*h* = −14→11
*T*_min_ = 0.244, *T*_max_ = 0.453	*k* = −6→6
5526 measured reflections	*l* = −20→20

### Refinement

**Table:**

Refinement on *F*^2^	Primary atom site location: structure-invariant direct methods
Least-squares matrix: full	Secondary atom site location: difference Fourier map
*R*[*F*^2^ > 2σ(*F*^2^)] = 0.025	Hydrogen site location: inferred from neighbouring sites
*wR*(*F*^2^) = 0.064	H-atom parameters constrained
*S* = 1.03	*w* = 1/[σ^2^(*F*_o_^2^) + (0.0308*P*)^2^ + 0.224*P*] where *P* = (*F*_o_^2^ + 2*F*_c_^2^)/3
2010 reflections	(Δ/σ)_max_ < 0.001
161 parameters	Δρ_max_ = 0.19 e Å^−^^3^
2 restraints	Δρ_min_ = −0.15 e Å^−^^3^

### Special details

**Table:**

Geometry. All esds (except the esd in the dihedral angle between two l.s. planes) are estimated using the full covariance matrix. The cell esds are taken into account individually in the estimation of esds in distances, angles and torsion angles; correlations between esds in cell parameters are only used when they are defined by crystal symmetry. An approximate (isotropic) treatment of cell esds is used for estimating esds involving l.s. planes.
Refinement. Refinement of F^2^ against ALL reflections. The weighted R-factor wR and goodness of fit S are based on F^2^, conventional R-factors R are based on F, with F set to zero for negative F^2^. The threshold expression of F^2^ > 2sigma(F^2^) is used only for calculating R-factors(gt) etc. and is not relevant to the choice of reflections for refinement. R-factors based on F^2^ are statistically about twice as large as those based on F, and R- factors based on ALL data will be even larger.

### Fractional atomic coordinates and isotropic or equivalent isotropic displacement parameters (Å^2^)

**Table:**

	*x*	*y*	*z*	*U*_iso_*/*U*_eq_	
C1	0.47072 (17)	0.1107 (4)	0.71936 (11)	0.0451 (5)	
H1	0.5182	−0.0211	0.7290	0.054*	
C2	0.41390 (18)	0.1315 (4)	0.64899 (11)	0.0538 (5)	
H2	0.4237	0.0140	0.6116	0.065*	
C3	0.34319 (17)	0.3250 (4)	0.63443 (11)	0.0514 (5)	
H3	0.3044	0.3374	0.5875	0.062*	
C4	0.33005 (16)	0.4994 (4)	0.68923 (12)	0.0495 (5)	
H4	0.2828	0.6311	0.6792	0.059*	
C5	0.38659 (15)	0.4814 (3)	0.75956 (11)	0.0428 (5)	
H5	0.3773	0.6012	0.7963	0.051*	
C6	0.45685 (14)	0.2859 (3)	0.77523 (9)	0.0338 (4)	
C7	0.50867 (14)	0.2546 (3)	0.85404 (9)	0.0346 (4)	
C8	0.59499 (14)	0.4187 (3)	0.88197 (9)	0.0336 (4)	
C9	0.63412 (15)	0.4204 (3)	0.96059 (10)	0.0359 (4)	
C10	0.71438 (15)	0.5983 (4)	0.98239 (10)	0.0410 (4)	
H10	0.7376	0.6093	1.0336	0.049*	
C11	0.75958 (15)	0.7561 (4)	0.93082 (11)	0.0411 (4)	
C12	0.72465 (16)	0.7507 (4)	0.85333 (10)	0.0428 (5)	
H12	0.7555	0.8556	0.8180	0.051*	
C13	0.64357 (15)	0.5853 (4)	0.83168 (10)	0.0389 (4)	
H13	0.6191	0.5830	0.7806	0.047*	
C14	0.88453 (18)	1.0924 (4)	0.91151 (13)	0.0564 (6)	
H14A	0.9237	1.0160	0.8708	0.085*	
H14B	0.9358	1.1940	0.9406	0.085*	
H14C	0.8241	1.1898	0.8904	0.085*	
Ni1	0.5000	0.0000	1.0000	0.03463 (12)	
N1	0.47005 (13)	0.0801 (3)	0.89494 (8)	0.0404 (4)	
H1A	0.4234	−0.0153	0.8712	0.048*	
O2	0.84011 (12)	0.9107 (3)	0.96004 (8)	0.0573 (4)	
O1	0.60041 (10)	0.2698 (2)	1.01257 (7)	0.0437 (3)	

### Atomic displacement parameters (Å^2^)

**Table:**

	*U*^11^	*U*^22^	*U*^33^	*U*^12^	*U*^13^	*U*^23^
C1	0.0554 (12)	0.0395 (11)	0.0399 (10)	0.0065 (10)	−0.0067 (9)	0.0015 (9)
C2	0.0702 (14)	0.0538 (14)	0.0369 (11)	−0.0003 (12)	−0.0086 (10)	−0.0047 (10)
C3	0.0531 (12)	0.0599 (14)	0.0401 (11)	−0.0120 (11)	−0.0150 (9)	0.0128 (10)
C4	0.0407 (11)	0.0483 (12)	0.0587 (13)	0.0024 (10)	−0.0116 (9)	0.0105 (11)
C5	0.0416 (10)	0.0390 (11)	0.0475 (11)	0.0029 (9)	−0.0031 (8)	−0.0009 (9)
C6	0.0330 (9)	0.0353 (10)	0.0329 (9)	−0.0041 (8)	−0.0017 (7)	0.0061 (8)
C7	0.0343 (9)	0.0371 (10)	0.0322 (9)	0.0030 (8)	0.0011 (7)	−0.0004 (8)
C8	0.0350 (10)	0.0356 (9)	0.0301 (9)	0.0030 (8)	−0.0002 (7)	0.0006 (7)
C9	0.0369 (10)	0.0369 (10)	0.0339 (9)	0.0031 (8)	0.0009 (8)	0.0010 (8)
C10	0.0449 (11)	0.0458 (11)	0.0320 (9)	−0.0028 (9)	−0.0037 (8)	−0.0015 (9)
C11	0.0365 (10)	0.0430 (11)	0.0439 (10)	−0.0048 (9)	0.0016 (8)	−0.0052 (9)
C12	0.0426 (10)	0.0463 (12)	0.0397 (10)	−0.0081 (9)	0.0045 (8)	0.0035 (9)
C13	0.0400 (10)	0.0433 (11)	0.0334 (10)	0.0005 (9)	0.0001 (8)	0.0030 (8)
C14	0.0523 (13)	0.0493 (12)	0.0680 (14)	−0.0138 (11)	0.0090 (11)	−0.0067 (11)
Ni1	0.0386 (2)	0.0381 (2)	0.02698 (17)	−0.00429 (15)	−0.00215 (12)	0.00705 (14)
N1	0.0476 (9)	0.0416 (9)	0.0316 (7)	−0.0113 (7)	−0.0048 (7)	0.0054 (7)
O2	0.0616 (9)	0.0619 (9)	0.0483 (8)	−0.0262 (8)	−0.0007 (7)	−0.0050 (7)
O1	0.0501 (8)	0.0482 (8)	0.0323 (6)	−0.0107 (6)	−0.0051 (5)	0.0080 (6)

### Geometric parameters (Å, º)

**Table:**

C1—C6	1.387 (3)	C9—C10	1.410 (3)
C1—C2	1.389 (3)	C10—C11	1.374 (3)
C1—H1	0.9300	C10—H10	0.9300
C2—C3	1.374 (3)	C11—O2	1.366 (2)
C2—H2	0.9300	C11—C12	1.405 (3)
C3—C4	1.369 (3)	C12—C13	1.369 (3)
C3—H3	0.9300	C12—H12	0.9300
C4—C5	1.385 (3)	C13—H13	0.9300
C4—H4	0.9300	C14—O2	1.424 (3)
C5—C6	1.383 (2)	C14—H14A	0.9600
C5—H5	0.9300	C14—H14B	0.9600
C6—C7	1.501 (2)	C14—H14C	0.9600
C7—N1	1.290 (2)	Ni1—N1	1.9118 (14)
C7—C8	1.439 (2)	Ni1—N1^i^	1.9118 (14)
C8—C13	1.407 (3)	Ni1—O1	1.9120 (13)
C8—C9	1.437 (2)	Ni1—O1^i^	1.9120 (13)
C9—O1	1.303 (2)	N1—H1A	0.8600
			
C6—C1—C2	120.12 (19)	C11—C10—H10	118.8
C6—C1—H1	119.9	C9—C10—H10	118.8
C2—C1—H1	119.9	O2—C11—C10	115.56 (16)
C3—C2—C1	120.3 (2)	O2—C11—C12	123.72 (17)
C3—C2—H2	119.9	C10—C11—C12	120.71 (17)
C1—C2—H2	119.9	C13—C12—C11	117.63 (17)
C4—C3—C2	119.73 (18)	C13—C12—H12	121.2
C4—C3—H3	120.1	C11—C12—H12	121.2
C2—C3—H3	120.1	C12—C13—C8	123.98 (16)
C3—C4—C5	120.59 (19)	C12—C13—H13	118.0
C3—C4—H4	119.7	C8—C13—H13	118.0
C5—C4—H4	119.7	O2—C14—H14A	109.5
C6—C5—C4	120.21 (18)	O2—C14—H14B	109.5
C6—C5—H5	119.9	H14A—C14—H14B	109.5
C4—C5—H5	119.9	O2—C14—H14C	109.5
C5—C6—C1	119.06 (16)	H14A—C14—H14C	109.5
C5—C6—C7	119.85 (16)	H14B—C14—H14C	109.5
C1—C6—C7	120.87 (16)	N1—Ni1—N1^i^	180.00 (2)
N1—C7—C8	122.72 (15)	N1—Ni1—O1	91.56 (6)
N1—C7—C6	116.88 (15)	N1^i^—Ni1—O1	88.44 (6)
C8—C7—C6	120.39 (15)	N1—Ni1—O1^i^	88.44 (6)
C13—C8—C9	117.89 (16)	N1^i^—Ni1—O1^i^	91.56 (6)
C13—C8—C7	119.90 (15)	O1—Ni1—O1^i^	180.0
C9—C8—C7	122.21 (16)	C7—N1—Ni1	130.26 (13)
O1—C9—C10	118.23 (15)	C7—N1—H1A	114.9
O1—C9—C8	124.53 (16)	Ni1—N1—H1A	114.9
C10—C9—C8	117.24 (16)	C11—O2—C14	118.83 (16)
C11—C10—C9	122.44 (17)	C9—O1—Ni1	128.17 (11)

Symmetry code: (i) −*x*+1, −*y*, −*z*+2.
